# Novel polymorphism at *ARHGAP24* gene and its association with growth traits in Hu sheep

**DOI:** 10.1080/10495398.2025.2513958

**Published:** 2025-06-11

**Authors:** Huili Shan, Liang yong Guo, Xin Huang, Xiaowei Zhang, Yongqing Jiang, Sangang He, Junfang Jiang

**Affiliations:** a Institute of Animal Husbandry and Veterinary, Zhejiang Academy of Agricultural Sciences, Hangzhou, Zhejiang, China; b Institute of Animal Science, Huzhou Agricultural Science and Technology Development Center, Huzhou, Zhejiang, China; c Zhejiang Animal Husbandry Technology Extension and Breeding Livestock & Poultry Testing Station, Hangzhou, Zhejiang, China

**Keywords:** Rho GTPase activating protein 24, association analysis, single-nucleotide polymorphism, haplotype, Hu sheep

## Abstract

It is essential to identify potential genes and genetic variants associated with growth traits to enhance sheep breeding. Previous studies have suggested that the Rho GTPase-activating protein 24 (*ARHGAP24*) gene may influence the growth of some animals. However, no association has been established between polymorphisms in the *ARHGAP24* gene and sheep growth traits. In this study, single-nucleotide polymorphisms (SNPs) in the *ARHGAP24* gene of Hu sheep were detected, and functional SNPs associated with Hu sheep growth traits were identified. Twenty-six SNPs were discovered in Hu sheep. Association analysis revealed NC_056059.1:g.455981A > G was significantly associated with average daily gain from six months to one year of age, while NC_056059.1:g.456083 A > G was significantly associated with average daily gain from weaning to six months of age. The relative fluorescence activity (firefly luciferase and sea kidney luciferase) of haplotype GGGG was significantly lower than that of haplotypes GGAA, AAAA and AAGG. Furthermore, *ARHGAP24* mRNA expression in the longissimus dorsi muscle of six-months-old sheep was significantly lower than birth sheep. The results showed that NC_056059.1:g.455981A > G and g NC_056059.1:g.456083A > G of *ARHGAP24* gene were related to some growth traits of Hu sheep, and *ARHGAP24* mRNA was differentially expressed in longissimus dorsi muscle of Hu sheep at different months of age, which could be used as a candidate gene for molecular marker-assisted selection of Hu sheep.

## Introduction

Hu sheep, a rare breed in China, are native to the Taihu Lake region and are renowned for their uniquely patterned lambskins. As one of the few breeds producing white lambskins, they are known as the ‘soft jewel’ in the international market.^1^ Sheep are highly adaptable, resistant to coarse feeding, and suitable for captive breeding. They exhibit year-round fertility, excellent maternal care, rapid growth, the ability to reproduce at six months and a lambing rate exceeding 200%. They are characterized by their large size, high meat production and flavourful, nutrient-rich meat. Lamb meat offers several advantages over other meats, including juiciness, low cholesterol and fat, high protein content and reduced odour.^2^ Despite these qualities, Hu sheep perform significantly differently in meat production compared to foreign meat breeds such as Dupo and German Merino sheep. Therefore, selecting and breeding Hu sheep with improved growth performance and meat production is essential.^3–5^

Growth traits are economically significant for sheep. Advances in molecular genetics have provided new opportunities to utilize genomics for identifying functional genes. In various species, quantitative trait loci (QTL) mapping and complex trait localization have been widely achieved using SNPs.^6^ The discovery of abundant SNPs has enhanced our understanding of the relationship between genomic variations and different traits, aided by modern sequencing technologies and bioinformatics tools.^7^

Rho GTPase-activating protein 24 (*ARHGAP24*) is a member of the Rho GTPase-activating proteins (Rho-GAPs) family and consists of 748 amino acids.^8^ Previous studies suggest that the *ARHGAP24* gene may influence proliferation, cell-cycle progression, apoptosis, migration and invasion of kidney cancer cells.^9^^,^^10^ Additionally, Uehara et al. demonstrated *ARHGAP24*’s role in ADP ribosylation factor 6-dependent pseudopod formation in human breast carcinoma cells.^11^ Hara et al. hypothesized that *ARHGAP24* contributes to astrocytoma progression by increasing Rac1 activity.^12^ The development of paclitaxel resistance has been linked to *ARHGAP24* downregulation in lung adenocarcinoma.^13^^,^^14^ Jin et al. reported that CBX3 upregulation promotes pulmonary adenocarcinoma cell growth by suppressing *ARHGAP24* and activating Rac1 signaling.^15^ Overexpression of *ARHGAP24* has been shown to regulate cellular function and apoptosis by modulating p53, p21 and Bax, thereby inhibiting colon cancer cell proliferation.^8^

Several studies have suggested that the *ARHGAP24* gene influences animal growth. Five candidate SNPs in *ARHGAP24* were associated with average daily gain (ADG) between 30 and 100 kg and days to 100 kg of body weight (D100) in Duroc male pigs.^16^ Additionally, D100 was associated with a candidate SNP within *ARHGAP24* in Yorkshire pigs.^1^ In Xiangsu hybrid pigs, the g.735313C > T locus in *ARHGAP24* was significantly associated with chest and abdominal circumferences.^18^ Xu et al. suggested that *ARHGAP24* polymorphisms may affect animal growth and development.^19^ In addition, rs335052970 was highlighted as a functional mutation for aggressive behavioural traits that changed the transcriptional activity of the *ARHGAP24* gene by affecting the binding of the transcription factor p53.^19^ Therefore, it is speculated that the SNP of the *ARHGAP24* gene is associated with the growth traits of Hu sheep.

No studies have reported associations between *ARHGAP24* gene variations and sheep growth traits. This study focused on Hu sheep and selected *ARHGAP24* as a candidate gene. SNPs in *ARHGAP24* were analysed through direct sequencing of PCR products to assess their association with Hu sheep growth traits. The study also examined expression differences in *ARHGAP24* gene during sheep growth, providing a theoretical basis for molecular breeding of mutton Hu sheep.

## Materials and methods

All animal-related procedures were reviewed and approved according to the Guidelines for the Care and Use of Animals of the Zhejiang Academy of Agricultural Sciences. The study was approved by the ethics committee of the Zhejiang Academy of Agricultural Sciences, Hangzhou, China (2021ZAASLA18).

### Animals, samples collection and DNA extraction

The study involved 308 Hu sheep (ewes) provided by Hangzhou Pangda Agricultural Development Co. The sheep were kept under uniform conditions. Hu sheep were 12 months old and non-pregnant. The experiment was conducted in autumn under a confined housing system. All sheep were managed under identical conditions and fed according to the NY/T 816-2021 Nutrient requirements of meat-type sheep and goat.

A 10 mL jugular blood sample was collected from each sheep, placed in EDTA anticoagulant tubes, and stored at −20 °C. Body weight and size measurements were recorded. Genomic DNA was extracted using TIANamp Blood DNA Kits (Tiangen Biochemical Technology, Beijing, China). After electrophoresis detection, the DNA was stored at −20 °C for further experiments.

### Primer design and PCR amplification

Table 1 shows the oligonucleotide primers designed to specifically amplify the *ARHGAP24* gene in Hu sheep (Ensembl Accession: 101119904). These primers were synthesized by Hangzhou Youkang Biotechnology Co., Ltd.

**Table 1.** Primers of *ARHGAP24* gene PCR amplification.

Primer name	Primer sequence (5’→3’)	Amplified region	Annealing degree	Amplification length
1F	GCTACATAGGAAAGTCCACCAG	Exon 1 and part of intron 1	61 °C	601 bp
1R	CCTACTGGTTCGCTATGATG			
2F	CAGGGTAGACAAGTGAACAAAACA	A partial region Intron 1	53 °C	476 bp
2R	GGAAATCATCTGACAATTCTGGC			
3F	GACACACCTTGGATGTTCACTG	Exon 2 and part of Intron 2	59 °C	665 bp
3R	GAAGTGGTTGGTTGTGAGC			
4F	CTGGTCTCTGGCTTCTTTG	Intron 8, Exon 9 and 3utr	61 °C	747 bp
4R	CTTAACAGTAGCATTATGGGAC			

Polymerase chain reaction (PCR) was performed in a 25 μL reaction mixture containing 1 μL of DNA, 12.5 μL of KOD One^™^ PCR Master Mix (TOYOBO, Japan), 11.1 μL of double-distilled water, and 0.2 μL of 10 μM forward and reverse primers. The PCR protocol included 35 cycles of denaturation at 98 °C for 10 s, annealing at 61 °C, 53 °C, 59 °C, and 61 °C for 30 s, extension at 68 °C for 30 s, and storage at 4 °C.

### PCR product sequencing and sequence analysis

Each sample was amplified using the primers listed in Table 1. Electrophoresis was performed on a 1.5% agarose gel. Samples corresponding to the target band were sent to Anhui General Biological Co. (Anhui, China) for Sanger sequencing using the Thermo Fisher ABI 3730XL system. The forward and reverse Sanger sequence plots for each individual were analysed using Mutation Surveyor 5.02 (Softgenetics, USA) to identify mutation locations and genotype.

### Effects of candidate SNPs on gene transcriptional activity

According to the results of association analysis between different genotypes and growth traits of Hu sheep. After organizing the wild-type and mutant haplotype sequences, they were submitted to General Biosystems (Anhui) for full-gene synthesis and subsequent cloning into the pGL4.10 vector. The luciferase expression vector was transfected into porcine kidney cells (PK15). After 24 h, the activity of firefly luciferase and Renilla luciferase was measured using a plate reader and the Dual-Luciferase^®^ Reporter Assay System (Promega, USA). The fluorescence ratio between the two was calculated.

### Differential expression of ARHGAP24 gene in longissimus dorsi muscle during the growth of Hu sheep

RNA from longissimus dorsi muscle in brith sheep(0-day), two-months-old sheep and six-month-old sheep was extracted using the RNeasy Plus Universal Mini Kit (QIAGEN, Germany). Reverse transcription was performed with the Polestar initial complementary DNA synthesis kit and genomic DNA removal (Tiosbio, China). KOD SYBR quantitative PCR (qPCR) Mix (TOYOBO, Japan) was used to detect and quantify *ARHGAP24* gene mRNA expression in longissimus dorsi muscle. The 18s gene was used as the reference for normalization. The reaction conditions were as follows: 95 °C for 10 s, 60 °C for 10 s, and 72 °C for 10 s. Relevant primer pairs are listed in Table 2.

**Table 2.** Primers for qPCR to the detection of *ARHGAP24* gene in the longissimus dorsi muscle.

Primers	Primer sequence (5’→3’)	Annealing degree	Amplification product length
ARHGAP24	F:GGGGCAGCTACAGAACAAGGAGA	60 °C	288 bp
	R:GTCTGGGCTTTCTCGAGTGCTTCA		
18s rRNA	F:GACACGGACAGGATTGACAGATT		267 bp
	R:GAGCCAGTCAGTGTAGCGCG		

### Data statistics and analysis

Individual SNPs were analysed using Sanger sequencing with Mutation Surveyor 5.02 (SoftGenetics, LLC, State College, PA, USA). Gene frequency, genotype frequency, site heterozygosity (He), and Chi-square (χ^2^) values for each SNP were calculated using PopGen32 software. The polymorphism information content (PIC) was determined using PIC_Calc 0.6 software.

The *ARHGAP24* gene was evaluated using two-way analysis of variance (ANOVA) for relative luciferase activity and reverse transcription-PCR. These analyses were performed with the IBM SPSS Statistics (SPSS, USA). A *P*-value <0.05 was considered statistically significant. Three biological replicates were conducted for each sample, and The relative quantification of mRNA in 9 samples was performed based on the 2^−ΔΔCT^method.^20^ Results are presented as mean ± standard deviation. Data plotting was performed using GraphPad Prism 8.0.

### Association analysis

The General Linear Model (GLM; SPSS) was used to analyse SNPs associated with body weight and body size traits in Hu sheep. Since all samples were female sheep raised on the same farm under identical feeding and management conditions, field and gender effects were excluded from the data modelling.

A *P*-value < 0.05 was considered statistically significant.

The model used for analysis was as follows:

Y=Xβ+e

In which, Y: the phenotypic values of body size traits and body weight traits;

Xβ: fixed effect of SNP genotype;

e: residual effect;

## Result

### Single-nucleotide polymorphism (SNP) identification

**Figure 1.**
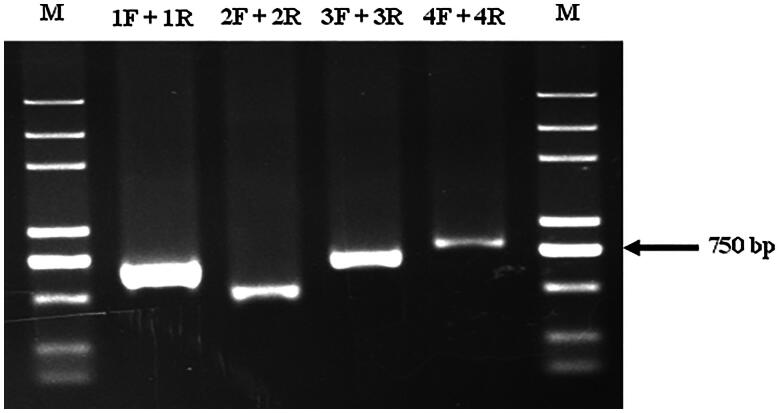
The results of PCR amplification (M: DNA MarkerDL5000).

The PCR product displayed high brightness, a single band, and no nonspecific amplification **(**Figure 1**).** The PCR result could be directly sequenced as the actual PCR product matched the expected size of the amplified fragment.

**Figure 2.**
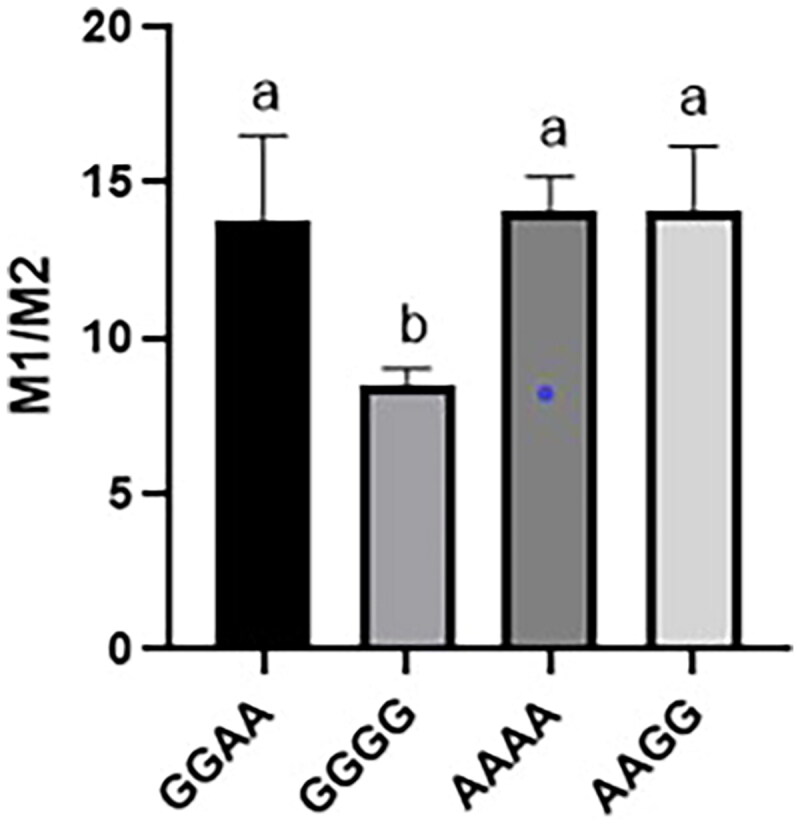
The detection results of luciferase activity in different groups. Notes: Different letters labeled on the bars indicate significant differences (*P* < 0.05).

**Figure 3.**
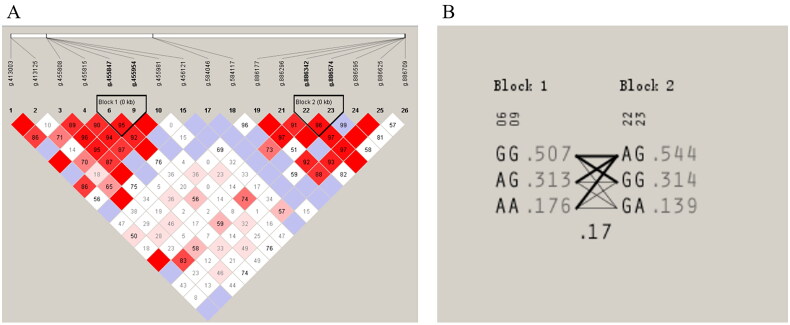
Linkage disequilibrium analysis ARHGAP24 gene. Notes: A. SNPs linkage disequilibrium analysis; B. Haplotype module analysis.

A total of 26 mutations were identified in the *ARHGAP24* gene of Hu sheep. Sequence analysis revealed two mutations with the first primer pair, 14 mutations with the second pair, two mutations with the third pair, and eight mutations with the fourth pair. These SNPs NC_056059.1: g.413003A > G, NC_056059.1:g.413125A > G,NC_056059.1:g.455808C > T, NC_056059.1:g.455815A > G,NC_056059.1:g.455820A > T, NC_056059.1:g.455847A > G, NC_056059.1:g.455865G > C, NC_056059.1:g.455922 G > A, NC_056059.1:g.455954G > A, NC_056059.1:g.455981A > G, NC_056059.1:g.456017G > A, NC_056059.1:g.456080A > G, NC_056059.1:g.456083A > G, NC_056059.1:g.456100A > G, NC_056059.1:g.456121C > T, and NC_056059.1:g.456146G > A are located in intron 1; NC_056059.1:g.584096T > C and NC_056059.1:g.584117A > G are in intron 2; NC_056059.1:g.886177A > G, NC_056059.1:g.886187A > G, NC_056059.1:g.886296 T > G, and NC_056059.1:g.886342A > G are in intron 8; NC_056059.1:g.886574G > A is in exon 9 (synonymous mutation); NC_056059.1:g.886595G > A and NC_056059.1:g.886625G > A are in the 3′ untranslated region (UTR); and NC_056059.1:g.886709G > A is downstream of the 3′ UTR (Table S1). Sanger sequencing figure in Supplement Figure 1, Figure 2, Figure 3.

### Population genetic analysis of SNPs of ARHGAP24

The effective allele number (Ne), average heterozygosity (He), and polymorphism information content (PIC) of the 26 SNP loci are presented in Table S2. Ne ranged from 1.05 to 2.00, with NC_056059.1:g.455847A > G and NC_056059.1:g.455865G > C having the highest values, and NC_056059.1:g.584096 T > C the lowest. The population’s average heterozygosity ranged from 0.04 to 0.50, with NC_056059.1:g.455847A > G, NC_056059.1:g.455865 G > C, and NC_056059.1:g.886342 A > G showing the highest values, and NC_056059.1:g.584096 T > C the lowest.

PIC values were classified as follows: 0.25 < PIC <0.50, moderately polymorphic; and PIC < 0.25, lowly polymorphic^21^. Eight loci (NC_056059.1:g.413003A > G, NC_056059.1:g.456121C > T, NC_056059.1:g.584096 T > C, gNC_056059.1:g.584117A > G, NC_056059.1:g.886187A > G, NC_056059.1:g.886296T > G, NC_056059.1:g.886574G > A, and NC_056059.1:g.886709 G > A) were lowly polymorphic, while the remaining 18 loci were moderately polymorphic (Table S3).

The results of the Hardy-Weinberg equilibrium test are shown in Table S3. A *P* < 0.05 indicated a population deviation from equilibrium. According to the χ^2^ test, the loci NC_056059.1:g.413003A > G, NC_056059.1:g.413125 A > G, NC_056059.1:g.456121C > T, NC_056059.1:g.584096T > C, gNC_056059.1:g.584117A > G, NC_056059.1:g.886177A > G, NC_056059.1:g.886296T > G, NC_056059.1:g.886342A > G, NC_056059.1:g.886574G > A, NC_056059.1:g.886595G > A, NC_056059.1:g.886625G > A, and NC_056059.1:g.886709G > A were in Hardy-Weinberg equilibrium, while the remaining 14 SNPs were not.

### Association analysis of ARHGAP24 gene polymorphism with growth traits

Association analysis revealed that NC_056059.1:g.455981A > G was significantly associated with average daily gain from six months to one year of age. The AA and AG genotypes significantly higher than the GG genotype (*P* < 0.05), while there was no significant difference between the AA and AG genotypes (*P* > 0.05). The NC_056059.1:g.456083A > G was significantly associated with average daily gain from weaning to six months of age. The GG genotype significantly higher than the AA genotype (*P* < 0.05), with no significant difference between the AG and GG genotypes (*P* > 0.05) **(**Table 3**)**.

**Table 3.** Association analysis between the *ARHGAP24* gene single nucleotide polymorphisms and growth traits in Hu sheep.

Loci name	Genetype	Body height (cm)	Circumference(cm)	Birth weight (kg)	Average daily gain before weaning (kg)	Weaning weight (kg)	Average daily gain from weaning to six months of age (kg)	six-month-old weight (kg)	Average daily gain from six months to one year of age (kg)	one-year-old weight (kg)
NC_056059.1:g.455981 A > G	AA (191)	75.03 ± 3.67	100.52 ± 7.29	3.03 ± 0.46	0.35 ± 0.13	19.05 ± 2.27	0.16 ± 0.02	38.09 ± 3.75	0.18 ± 0.06^a^	64.42 ± 9.96
	AG (89)	74.79 ± 3.20	99.93 ± 6.51	3.04 ± 0.44	0.36 ± 0.14	18.69 ± 2.25	0.16 ± 0.02	38.09 ± 3.63	0.18 ± 0.05^a^	63.13 ± 8.90
	GG (28)	73.27 ± 4.09	99.35 ± 6.65	3.07 ± 0.46	0.29 ± 0.09	19.59 ± 2.96	0.16 ± 0.02	38.95 ± 5.20	0.15 ± 0.04^b^	64.56 ± 9.54
	*P*	0.06	0.63	0.92	0.08	0.18	0.46	0.52	0.02	0.56
NC_056059.1:g.456083 A > G	AA (219)	74.71 ± 3.68	100.29 ± 6.89	3.04 ± 0.45	0.34 ± 0.13	19.07 ± 2.47	0.15 ± 0.02^b^	38.07 ± 3.71	0.18 ± 0.05	64.38 ± 9.06
	AG (56)	74.72 ± 2.99	100.21 ± 5.70	2.95 ± 0.39	0.35 ± 0.14	18.55 ± 1.63	0.16 ± 0.02^ab^	37.82 ± 3.58	0.18 ± 0.05	63.16 ± 8.82
	GG (33)	75.59 ± 4.00	99.92 ± 9.55	3.15 ± 0.54	0.36 ± 0.13	19.28 ± 2.40	0.17 ± 0.03^a^	39.44 ± 5.06	0.17 ± 0.06	63.46 ± 11.71
	*P*	0.42	0.96	0.15	0.55	0.23	0.03	0.12	0.76	0.65

Note: Data with the different superscript letters within the same column in the same location are significantly different (*P* < 0.05).

### ARHGAP24 gene transcriptional activity analysis of candidate SNPs

In Hu sheep, NC_056059.1:g.455981A > G was significantly associated with average daily gain from six months to one year of age. The NC_056059.1:g.456083A > G was significantly associated with weight gain from weaning to six-months-old weight. Further in vitro experiments were conducted using the four haplotypes GGAA, GGGG, AAAA, and AAGG of NC_056059.1:g.455981A > G and NC_056059.1:g.456083A > G. Recombinant plasmids were transfected, and measurements were taken after 24 h. The results showed that the relative fluorescence activity of the GGGG haplotype was significantly lower than that of GGAA, AAAA, and AAGG among the four haplotypes (Figure 2).

### Linkage disequilibrium and haplotype block analyses

Haploview software was used to analyse the linkage disequilibrium of all SNP loci and construct haplotypes, as shown in Figure 3. Two significant linkage blocks were identified among the 26 SNP loci. Block 1, formed by NC_056059.1:g.455847A > G and NC_056059.1:g.455954G > A, produced three haplotypes: GG, AG, and AA, with frequencies of 0.507, 0.313, and 0.176, respectively. Block 2, formed by NC_056059.1:g.886342A > G and NC_056059.1:g.886574G > A, produced three haplotypes: AG, GG, and GA, with frequencies of 0.544, 0.314, and 0.318, respectively.

### Differential expression of ARHGAP24 mRNA in longissimus dorsi muscle during the growth of Hu sheep

**Figure 4.**
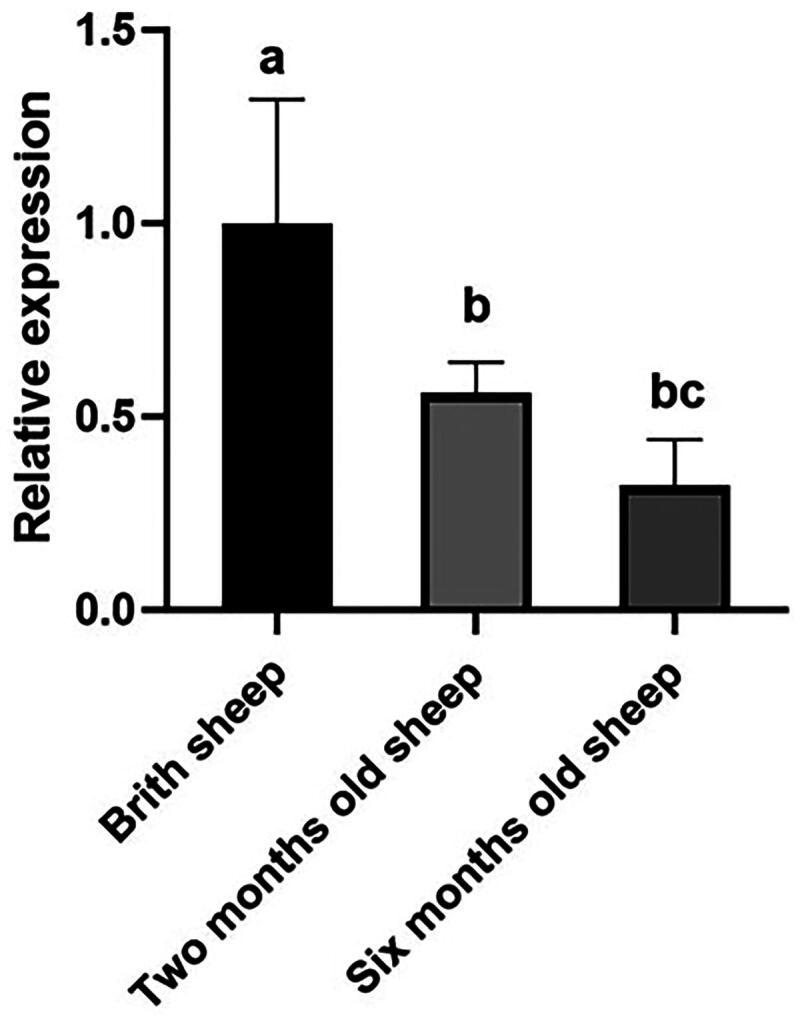
Detection of ARHGAP24 mRNA at different ages. Notes: Different letters labelled on the bars indicate significant differences (*P* < 0.05).

The expression of *ARHGAP24* mRNA in the longissimus dorsi muscle of six-month-old sheep was significantly lower than birth sheep **(**Figure 4**)**.

## Discussion

The Rho family consists of Ras-related small actin-based Rho GTPases, which act as molecular switches to regulate various cellular functions, including cytoskeletal organization and gene transcription. Rho-GAPs, the most abundant class of Rho GTPase regulators in eukaryotes, are essential for cytoskeletal organization, growth, differentiation, neural development, and synaptic functions.^22^^,^^23^ Recent studies have shown that ARHGAP21 influences body weight and insulin energy metabolism in mice.^24–26^ Genetic variation can affect the phenotypic traits of animals by altering gene expression and function.^27^^,^^28^

In this study, 26 SNPs were identified in the *ARHGAP24* gene of Hu sheep, including g.474408G > A, which was located in exon 9 and classified as a synonymous mutation. Generally, higher polymorphism information content (PIC) indicates greater genetic diversity in a population. Several SNPs, such as NC_056059.1:g.413125A > G, NC_056059.1:g.455808 C > T, NC_056059.1:g.455815A > G, NC_056059.1:g.455820A > T, NC_056059.1:g.455847A > G, NC_056059.1:g.455865G > C, NC_056059.1:g.455922G > A, NC_056059.1:g.455954G > A, NC_056059.1:g.455981 A > G, NC_056059.1:g.456017G > A, NC_056059.1:g.456080 A > G, NC_056059.1:g.456083 A > G, NC_056059.1:g.456100A > G, and NC_056059.1:g.456146 G > A, exhibited moderate polymorphism (0.25 < PIC < 0.50), indicating their selection potential. This suggests that the genetic resources of the population are well-suited for survival and reproduction in the current breeding environment.

Hardy–Weinberg equilibrium assumes a naturally occurring population with completely random mating, and the genetic equilibrium of the population is assessed using a chi-square test. According to the χ^2^ test results, loci NC_056059.1:g.413003A > G, NC_056059.1:g.413125 A > G, NC_056059.1:g.456121C > T, NC_056059.1:g.584096T > C, NC_056059.1:g.584117A > G, NC_056059.1:g.886177A > G, NC_056059.1:g.886296T > G, NC_056059.1:g.886342A > G, NC_056059.1:g.886574G > A, NC_056059.1:g.886595G > A, NC_056059.1:g.886625G > A, and NC_056059.1:g.886709G > A in the Hu sheep population were in Hardy-Weinberg equilibrium, while the remaining 14 SNPs deviated from equilibrium. Hardy-Weinberg disequilibrium is common in sheep breeding programs focused on production performance and physical traits. The primary cause of such deviations is the excess of homozygotes due to inbreeding.^29^

Polymorphisms in the *ARHGAP24* gene have been shown to influence growth traits in pigs and the progression of chronic hepatitis B virus infection in the Han Chinese population.^16^^,^^17^^,^^30^ Previous studies revealed that the g.735313C > T locus in *ARHGAP24* is significantly associated with chest and abdominal circumference in Xiangsu hybrid pigs. Individuals with the TT genotype exhibit greater chest and abdominal circumferences than those with the CT genotype.^18^ *ARHGAP24* polymorphisms may affect growth and development.^19^ In this study, two SNPs, NC_056059.1:g.455981A > G was significantly associated with average daily gain from six months to one year of age, while NC_056059.1:g.456083A > G was significantly associated with average daily gain from weaning to six months of age.

Among the four haplotypes (GGAA, GGGG, AAAA, and AAGG) of NC_056059.1: g.455981A > G and NC_056059.1:g.456083A > G, the relative fluorescence activity of GGGG was significantly lower than that of GGAA, AAAA, and AAGG (*P* < 0.05). As there was no significant difference between GGAA and AAAA, the first G allele may represent the primary effector gene. These findings suggest that the impact of NC_056059.1:g.455981A > G on transcriptional activity may be greater than that of NC_056059.1:g.456083A > G. Studies have shown that introns can directly and indirectly affect gene expression, mainly by changing splicing patterns and regulatory elements.^31^ SNP located in introns may influence gene expression, mRNA transport, and mRNA aging, thereby exerting post-transcriptional regulatory effects.^32^ Further research could focus on elucidating the molecular mechanisms by which the *ARHGAP24* gene regulates developmental traits in Hu sheep.

We concluded that *ARHGAP24* gene mutations are associated with Hu sheep growth traits based on prior association analyses and our data. Thus, *ARHGAP24* can be considered a candidate gene for growth traits in Hu sheep. Therefore, variations in the expression of candidate genes in the longissimus dorsi muscle were examined. Expression levels of *ARHGAP24* in six-month-old sheep were significantly lower than in brith sheep, that *ARHGAP24* may involved in the development of skeletal muscles. Consequently, the *ARHGAP24* gene could serve as a molecular marker for marker-assisted selection in sheep breeding, reducing the breeding cycle and advancing sheep industry development.

This study was limited to exon 1, part of intron 1, exon 2, part of intron 2, intron 8, exon 9, and the 3′ UTR. Therefore, detecting and analyzing SNPs in other regions of the *ARHGAP24* gene may help uncover its polymorphisms and precise effects.

## Conclusions

This study used Hu sheep as experimental material and selected *ARHGAP24* as a candidate gene. SNPs were screened using direct sequencing and sequence analysis. Two SNPs, NC_056059.1:g.455981A > G and NC_056059.1:g.456083A > G, were significantly associated with Hu sheep growth traits. The transcriptional activity of the candidate SNPs was analysed using a dual-luciferase assay. The results suggest that these SNPs could serve as important molecular markers for growth traits in sheep. This study provides novel insights into the genetic functions of *ARHGAP24* in Hu sheep.

## Supplementary Material

Table S2 Genetic Parameters of ARHGAP24 gene of Hu sheep.doc

Table S1 SNPs and mutation types and methods of ARHGAP24 gene of Hu sheep.doc

Supplement Figure 1.jpg

Supplement Figure 3.png

Supplement Figure 2.jpg

Table S3 Population Genetic Analysis of ARHGAP24 gene of Hu sheep.doc

Sanger sequencing results.zip

## Data Availability

Data included in article/supplementary material in article.
