# Supplementary material for: Novel polymorphism at ARHGAP24 gene and its association with growth traits in Hu sheep
Source: Anim Biotechnol. 2025 Jun 11;36(1):2513958. doi: 10.1080/10495398.2025.2513958 (PMC12674321; doi:10.1080/10495398.2025.2513958)
Supplement: Table S2 Genetic Parameters of ARHGAP24 gene of Hu sheep.doc [file LABT_A_2513958_SM5817.doc]

Table S2 Genetic Parameters of ARHGAP24 gene of Hu sheep

| Primer name | Loci name | Ne | He |
| --- | --- | --- | --- |
| 1F＋1R | NC_056059.1:g.413003A＞G | 1.07 | 0.07 |
|  | NC_056059.1:g.413125A＞G | 1.50 | 0.33 |
| 2F＋2R | NC_056059.1:g.455808C＞T | 2.00 | 0.50 |
|  | NC_056059.1:g.455815A＞G | 1.42 | 0.29 |
|  | NC_056059.1:g.455820A＞T | 1.43 | 0.30 |
|  | NC_056059.1:g.455847A＞G | 2.00 | 0.50 |
|  | NC_056059.1:g.455865G＞C | 2.00 | 0.50 |
|  | NC_056059.1:g.455922G＞A | 1.45 | 0.31 |
|  | NC_056059.1:g.455954G＞A | 1.42 | 0.29 |
|  | NC_056059.1:g.455981A＞G | 1.56 | 0.36 |
|  | NC_056059.1:g.456017G＞A | 1.52 | 0.34 |
|  | NC_056059.1:g.456080A＞G | 1.54 | 0.35 |
|  | NC_056059.1:g.456083A＞G | 1.47 | 0.32 |
|  | NC_056059.1:g.456100A＞G | 1.56 | 0.36 |
|  | NC_056059.1:g.456121C＞T | 1.06 | 0.06 |
|  | NC_056059.1:g.456146G＞A | 1.72 | 0.42 |
| 3F＋3R | NC_056059.1:g.584096T＞C | 1.05 | 0.04 |
|  | NC_056059.1:g.584117A＞G | 1.06 | 0.06 |
| 4F＋4R | NC_056059.1:g.886177A＞G | 1.49 | 0.33 |
|  | NC_056059.1:g.886187A＞G | 1.18 | 0.15 |
|  | NC_056059.1:g.886296T＞G | 1.29 | 0.23 |
|  | NC_056059.1:g.886342A＞G | 1.98 | 0.50 |
|  | NC_056059.1:g.886574G＞A | 1.32 | 0.24 |
|  | NC_056059.1:g.886595G＞A | 1.49 | 0.33 |
|  | NC_056059.1:g.886625G＞A | 1.50 | 0.33 |
|  | NC_056059.1:g.886709G＞A | 1.08 | 0.07 |
