# Supplementary material for: Novel polymorphism at ARHGAP24 gene and its association with growth traits in Hu sheep
Source: Anim Biotechnol. 2025 Jun 11;36(1):2513958. doi: 10.1080/10495398.2025.2513958 (PMC12674321; doi:10.1080/10495398.2025.2513958)
Supplement: Table S1 SNPs and mutation types and methods of ARHGAP24 gene of Hu sheep.doc [file LABT_A_2513958_SM5816.doc]

Table S1 SNPs and mutation types and methods of ARHGAP24 gene of Hu sheep

| Primer name | Loci name | Mutagenesis | Type of substitution | position |
| --- | --- | --- | --- | --- |
| 1F＋1R | NC_056059.1:g.413003A＞G | A＞G | transition | Intron1 |
|  | NC_056059.1:g.413125A＞G | A＞G | transition | Intron1 |
| 2F＋2R | NC_056059.1:g.455808C＞T | C＞T | transition | Intron1 |
|  | NC_056059.1:g.455815A＞G | A＞G | transition | Intron1 |
|  | NC_056059.1:g.455820A＞T | A＞T | transversion | Intron1 |
|  | NC_056059.1:g.455847A＞G | A＞G | transition | Intron1 |
|  | NC_056059.1:g.455865G＞C | G＞C | transversion | Intron1 |
|  | NC_056059.1:g.455922G＞A | G＞A | transition | Intron1 |
|  | NC_056059.1:g.455954G＞A | G＞A | transition | Intron1 |
|  | NC_056059.1:g.455981A＞G | A＞G | transition | Intron1 |
|  | NC_056059.1:g.456017G＞A | G＞A | transition | Intron1 |
|  | NC_056059.1:g.456080A＞G | A＞G | transition | Intron1 |
|  | NC_056059.1:g.456083A＞G | A＞G | transition | Intron1 |
|  | NC_056059.1:g.456100A＞G | A＞G | transition | Intron1 |
|  | NC_056059.1:g.456121C＞T | C＞T | transition | Intron1 |
|  | NC_056059.1:g.456146G＞A | G＞A | transition | Intron1 |
| 3F＋3R | NC_056059.1:g.584096T＞C | T＞C | transition | Intron2 |
|  | NC_056059.1:g.584117A＞G | A＞G | transition | Intron2 |
| 4F＋4R | NC_056059.1:g.886177A＞G | A＞G | transition | Intron8 |
|  | NC_056059.1:g.886187A＞G | A＞G | transition | Intron8 |
|  | NC_056059.1:g.886296T＞G | T＞G | transversion | Intron8 |
|  | NC_056059.1:g.886342A＞G | A＞G | transition | Intron8 |
|  | NC_056059.1:g.886574G＞A | G＞A | transition | Exon9(synonymous mutation) |
|  | NC_056059.1:g.886595G＞A | G＞A | transition | 3UTR |
|  | NC_056059.1:g.886625G＞A | G＞A | transition | 3UTR |
|  | NC_056059.1:g.886709G＞A | G＞A | transition | 3UTR downstream |
