# Supplementary figures and images for: Novel polymorphism at ARHGAP24 gene and its association with growth traits in Hu sheep
Source: Anim Biotechnol. 2025 Jun 11;36(1):2513958. doi: 10.1080/10495398.2025.2513958 (PMC12674321; doi:10.1080/10495398.2025.2513958)

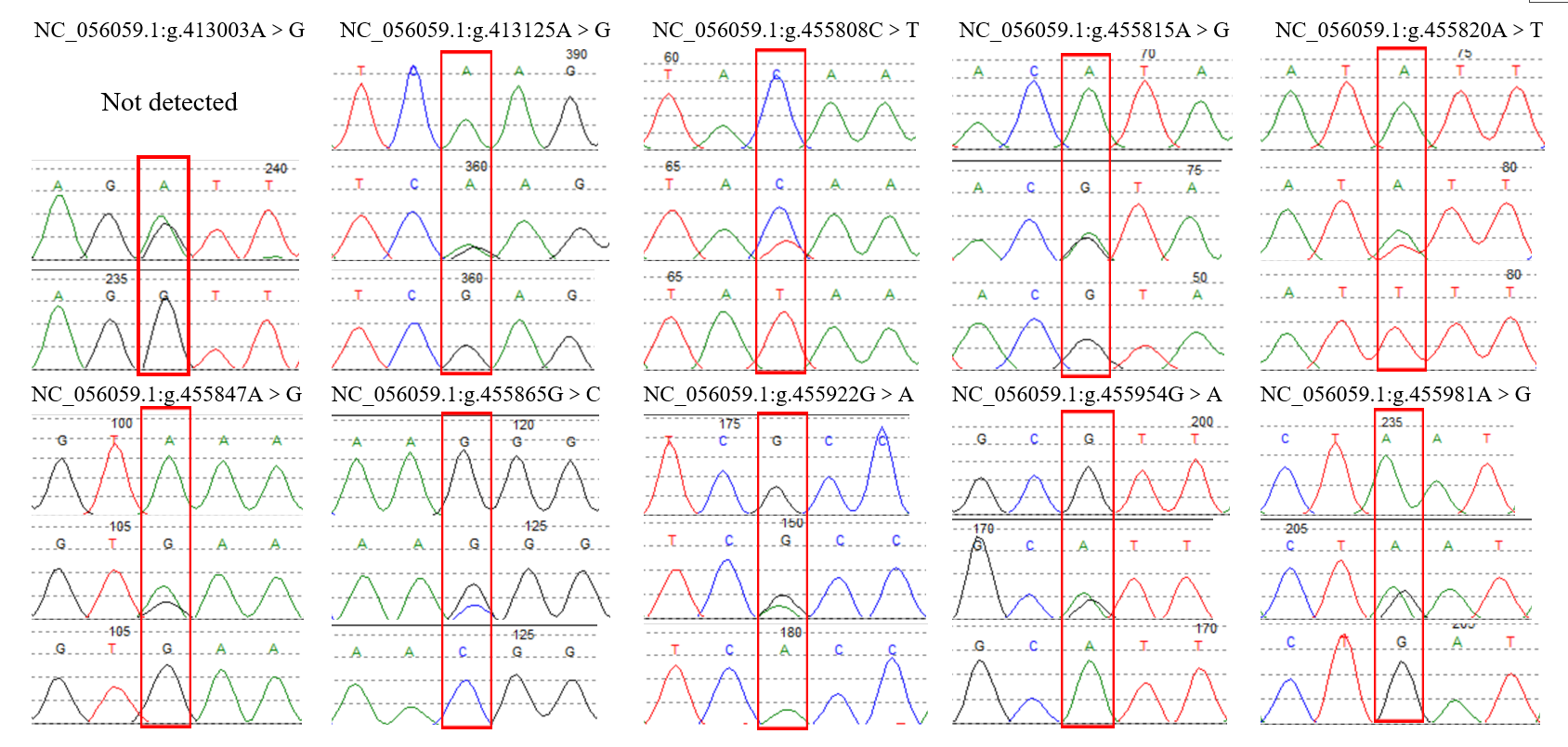

Supplement: Supplement Figure 1.jpg [file LABT_A_2513958_SM5815.jpg]

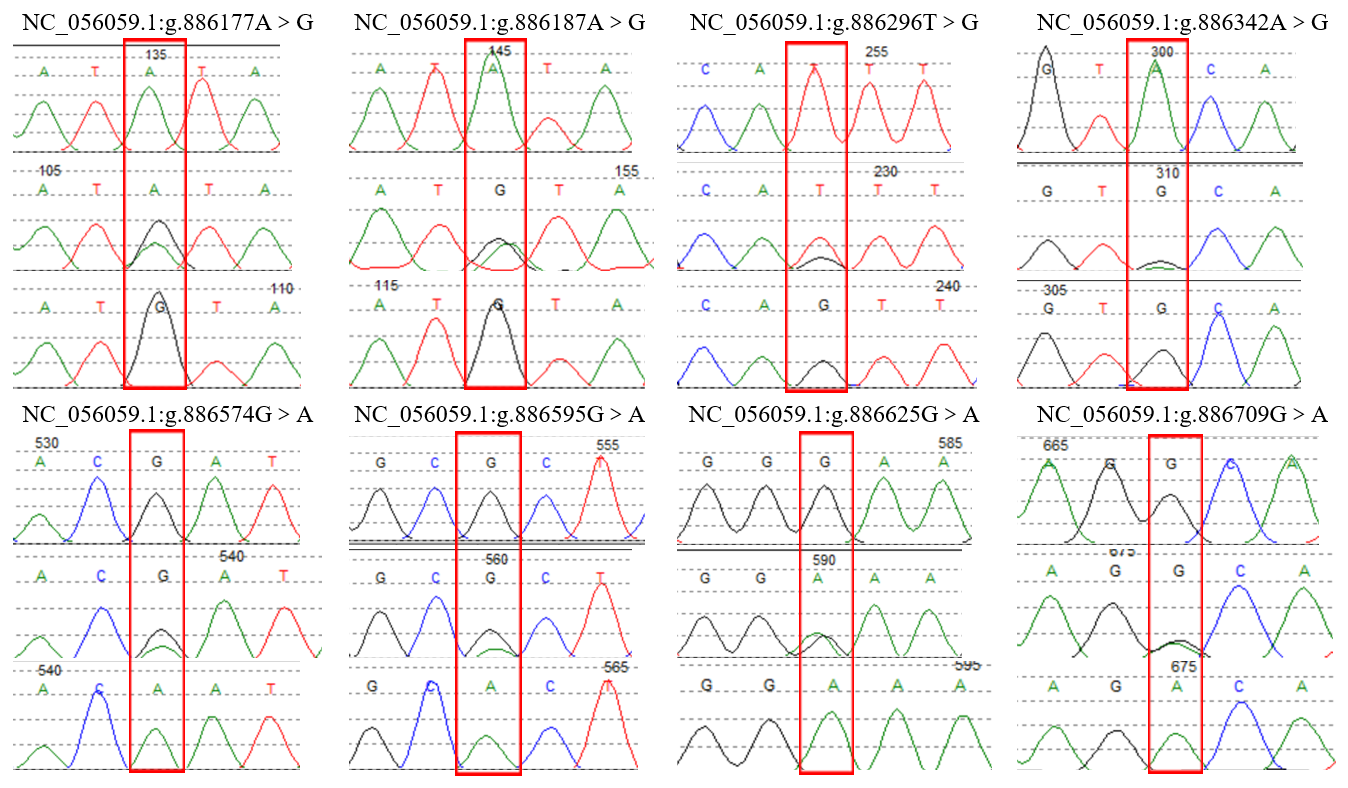

Supplement: Supplement Figure 3.png [file LABT_A_2513958_SM5814.png]

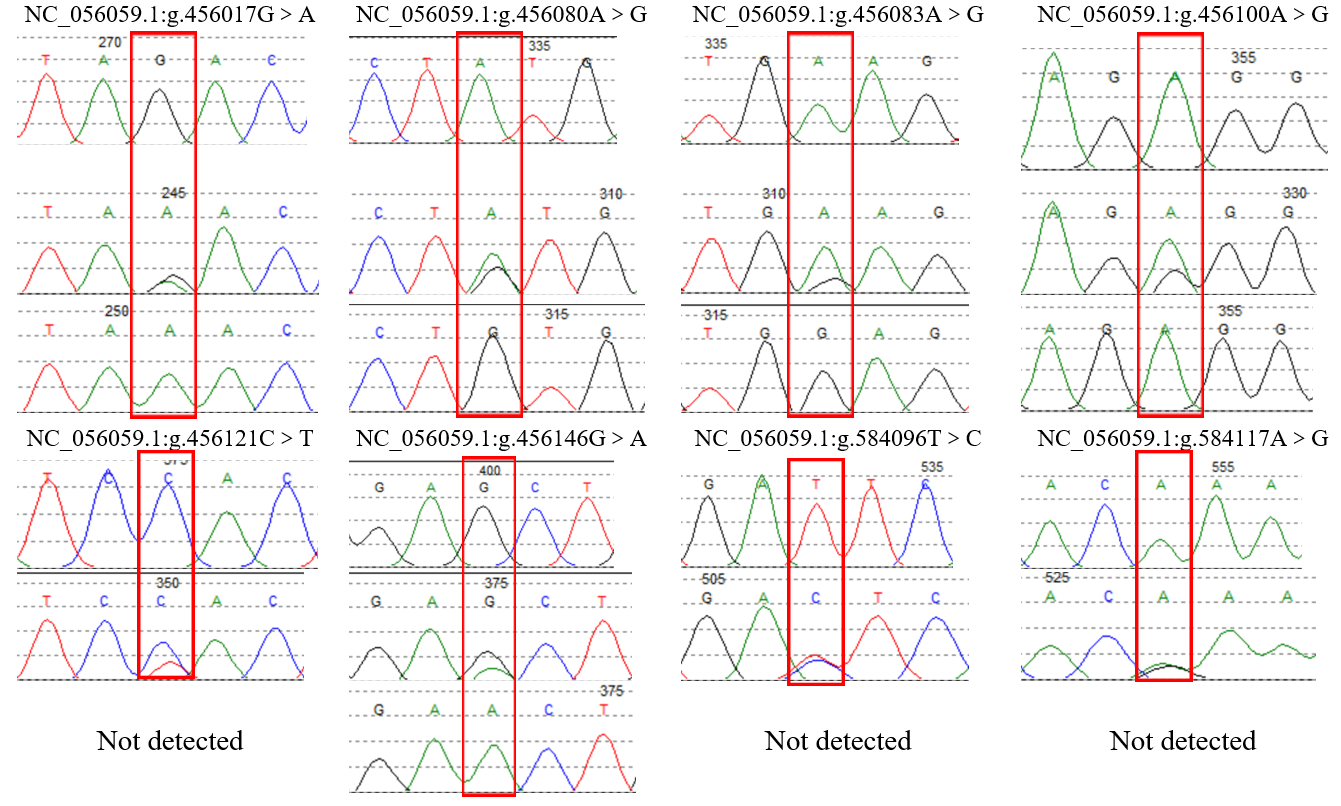

Supplement: Supplement Figure 2.jpg [file LABT_A_2513958_SM5813.jpg]
