# Supplementary material for: Novel polymorphism at ARHGAP24 gene and its association with growth traits in Hu sheep
Source: Anim Biotechnol. 2025 Jun 11;36(1):2513958. doi: 10.1080/10495398.2025.2513958 (PMC12674321; doi:10.1080/10495398.2025.2513958)
Supplement: Table S3 Population Genetic Analysis of ARHGAP24 gene of Hu sheep.doc [file LABT_A_2513958_SM5812.doc]

Table S3 Population Genetic Analysis of ARHGAP24 gene of Hu sheep

| Primer name | Loci name | Genetype | Numbers | Genetype frequencies | Allele | Allele frequencies | PIC | χ2 | *P* |
| --- | --- | --- | --- | --- | --- | --- | --- | --- | --- |
| 1F＋1R | NC_056059.1:g.413003A＞G | AA | 0 | 0.00 | A | 0.04 | 0.07 | 0.40 | 0.53 |
|  |  | AG | 22 | 0.07 |  |  |  |  |  |
|  |  | GG | 286 | 0.93 | G | 0.96 |  |  |  |
|  | NC_056059.1:g.413125A＞G | AA | 11 | 0.04 | A | 0.21 | 0.28 | 0.94 | 0.33 |
|  |  | AG | 109 | 0.35 |  |  |  |  |  |
|  |  | GG | 188 | 0.61 | G | 0.79 |  |  |  |
| 2F＋2R | NC_056059.1:g.455808C＞T | CC | 89 | 0.29 | C | 0.50 | 0.38 | 8.95 | 0.00 |
|  |  | TC | 128 | 0.41 |  |  |  |  |  |
|  |  | TT | 91 | 0.30 | T | 0.50 |  |  |  |
|  | NC_056059.1:g.455815A＞G | AA | 214 | 0.69 | A | 0.82 | 0.25 | 5.93 | 0.01 |
|  |  | AG | 78 | 0.26 |  |  |  |  |  |
|  |  | GG | 16 | 0.05 | G | 0.18 |  |  |  |
|  | NC_056059.1:g.455820A＞T | AA | 224 | 0.73 | A | 0.82 | 0.25 | 50.89 | 0.00 |
|  |  | AT | 55 | 0.18 |  |  |  |  |  |
|  |  | TT | 29 | 0.09 | T | 0.18 |  |  |  |
|  | NC_056059.1:g.455847A＞G | AA | 86 | 0.28 | A | 0.49 | 0.37 | 8.24 | 0.00 |
|  |  | AG | 129 | 0.42 |  |  |  |  |  |
|  |  | GG | 93 | 0.30 | G | 0.51 |  |  |  |
|  | NC_056059.1:g.455865G＞C | GG | 95 | 0.31 | G | 0.50 | 0.38 | 15.23 | 0.00 |
|  |  | GC | 120 | 0.39 |  |  |  |  |  |
|  |  | CC | 93 | 0.30 | C | 0.50 |  |  |  |
|  | NC_056059.1:g.455922G＞A | GG | 221 | 0.72 | G | 0.81 | 0.26 | 56.85 | 0.00 |
|  |  | GA | 55 | 0.18 |  |  |  |  |  |
|  |  | AA | 32 | 0.10 | A | 0.19 |  |  |  |
|  | NC_056059.1:g.455954G＞A | GG | 214 | 0.69 | G | 0.82 | 0.25 | 7.48 | 0.00 |
|  |  | GA | 77 | 0.25 |  |  |  |  |  |
|  |  | AA | 17 | 0.06 | A | 0.18 |  |  |  |
|  | NC_056059.1:g.455981A＞G | AA | 191 | 0.62 | A | 0.76 | 0.29 | 12.21 | 0.00 |
|  |  | AG | 89 | 0.29 |  |  |  |  |  |
|  |  | GG | 28 | 0.09 | G | 0.24 |  |  |  |
|  | NC_056059.1:g.456017G＞A | GG | 209 | 0.68 | G | 0.78 | 0.28 | 47.29 | 0.00 |
|  |  | GA | 64 | 0.21 |  |  |  |  |  |
|  |  | AA | 35 | 0.11 | A | 0.22 |  |  |  |
|  | NC_056059.1:g.456080A＞G | AA | 203 | 0.66 | A | 0.77 | 0.29 | 36.12 | 0.00 |
|  |  | AG | 71 | 0.23 |  |  |  |  |  |
|  |  | GG | 34 | 0.11 | G | 0.23 |  |  |  |
|  | NC_056059.1:g.456083A＞G | AA | 219 | 0.71 | A | 0.80 | 0.27 | 56.96 | 0.00 |
|  |  | AG | 56 | 0.18 |  |  |  |  |  |
|  |  | GG | 33 | 0.11 | G | 0.20 |  |  |  |
|  | NC_056059.1:g.456100A＞G | AA | 194 | 0.63 | A | 0.76 | 0.30 | 21.77 | 0.00 |
|  |  | AG | 82 | 0.27 |  |  |  |  |  |
|  |  | GG | 32 | 0.10 | G | 0.24 |  |  |  |
|  | NC_056059.1:g.456121C＞T | CC | 289 | 0.94 | C | 0.97 | 0.06 | 0.30 | 0.59 |
|  |  | CT | 19 | 0.06 |  |  |  |  |  |
|  |  | TT | 0 | 0.00 | T | 0.03 |  |  |  |
|  | NC_056059.1:g.456146G＞A | GG | 196 | 0.64 | G | 0.70 | 0.33 | 143.82 | 0.00 |
|  |  | GA | 41 | 0.13 |  |  |  |  |  |
|  |  | AA | 71 | 0.23 | A | 0.30 |  |  |  |
| 3F＋3R | NC_056059.1:g.584096T＞C | TT | 294 | 0.95 | T | 0.98 | 0.04 | 0.15 | 0.69 |
|  |  | TC | 14 | 0.05 |  |  |  |  |  |
|  |  | CC | 0 | 0.00 | C | 0.02 |  |  |  |
|  | NC_056059.1:g.584117A＞G | AA | 291 | 0.94 | A | 0.97 | 0.06 | 2.40 | 0.12 |
|  |  | AG | 17 | 0.05 |  |  |  |  |  |
|  |  | GG | 0 | 0.00 | G | 0.03 |  |  |  |
| 4F＋4R | NC_056059.1:g.886177A＞G | AA | 190 | 0.62 | A | 0.79 | 0.28 | 1.24 | 0.26 |
|  |  | AG | 108 | 0.35 |  |  |  |  |  |
|  |  | GG | 10 | 0.03 | G | 0.21 |  |  |  |
|  | NC_056059.1:g.886187A＞G | AA | 12 | 0.04 | A | 0.08 | 0.14 | 59.38 | 0.00 |
|  |  | AG | 26 | 0.08 |  |  |  |  |  |
|  |  | GG | 270 | 0.88 | G | 0.92 |  |  |  |
|  | NC_056059.1:g.886296T＞G | TT | 234 | 0.76 | T | 0.87 | 0.20 | 0.19 | 0.66 |
|  |  | TG | 68 | 0.22 |  |  |  |  |  |
|  |  | GG | 6 | 0.02 | G | 0.13 |  |  |  |
|  | NC_056059.1:g.886342A＞G | AA | 90 | 0.29 | A | 0.55 | 0.37 | 0.22 | 0.64 |
|  |  | AG | 157 | 0.51 |  |  |  |  |  |
|  |  | GG | 61 | 0.20 | G | 0.45 |  |  |  |
|  | NC_056059.1:g.886574G＞A | GG | 226 | 0.73 | G | 0.86 | 0.21 | 0.26 | 0.61 |
|  |  | GA | 77 | 0.25 |  |  |  |  |  |
|  |  | AA | 5 | 0.02 | A | 0.14 |  |  |  |
|  | NC_056059.1:g.886595G＞A | GG | 188 | 0.61 | G | 0.79 | 0.28 | 2.32 | 0.13 |
|  |  | GA | 111 | 0.36 |  |  |  |  |  |
|  |  | AA | 9 | 0.03 | A | 0.21 |  |  |  |
|  | NC_056059.1:g.886625G＞A | GG | 190 | 0.62 | G | 0.79 | 0.28 | 0.31 | 0.58 |
|  |  | GA | 106 | 0.34 |  |  |  |  |  |
|  |  | AA | 12 | 0.04 | A | 0.21 |  |  |  |
|  | NC_056059.1:g.886709G＞A | GG | 286 | 0.93 | G | 0.96 | 0.07 | 0.91 | 0.34 |
|  |  | GA | 21 | 0.07 |  |  |  |  |  |
|  |  | AA | 1 | 0.00 | A | 0.04 |  |  |  |
